# First-year drug therapy of new-onset rheumatoid and undifferentiated arthritis: a nationwide register-based study

**DOI:** 10.1186/s41927-020-00127-6

**Published:** 2020-07-03

**Authors:** Paula Muilu, Vappu Rantalaiho, Hannu Kautiainen, Lauri J. Virta, Johan G. Eriksson, Kari Puolakka

**Affiliations:** 1 Department of Medicine, Tampere University Hospital, Teiskontie 35, 33520 Tampere, Finland; 2 Centre for Rheumatic Diseases, Tampere University Hospital, Tampere, Finland; 3 Faculty on Medicine and Health Technology, Tampere University, Tampere, Finland; 4 Primary Health Care Unit, Kuopio University Hospital, Kuopio, Finland; 5 Folkhälsan Research Center, Helsinki, Finland; 6 Research Department, Social Insurance Institution of Finland, Turku, Finland; 7 Department of General Practice and Primary Health Care, University of Helsinki, Helsinki, Finland; 8 Department of Obstetrics and Gynecology, National University Singapore, Yong Loo Lin School of Medicine, Singapore, Singapore; 9 Singapore Institute for Clinical Sciences (SICS), Agency for Science, Technology and Research (A*STAR), Singapore, Singapore; 10 Department of Medicine, South Karelia Central Hospital, Lappeenranta, Finland

**Keywords:** Rheumatoid arthritis, Undifferentiated arthritis, Antirheumatic drugs, Disease modifying, Biologic therapy

## Abstract

**Background:**

In this retrospective cohort study, we evaluated the drug therapies used for early rheumatoid (RA) and undifferentiated (UA) arthritis patients.

**Methods:**

From a nationwide register maintained by the Social Insurance Institution, information on sex, date of birth, and date of special medicine reimbursement decision for all new Finnish RA and UA patients between 2011 and 14 were collected, and their DMARD (Disease Modifying Antirheumatic Drug) purchases during the first year after the diagnosis were analyzed.

**Results:**

A total of 7338 patients with early RA (67.3% female, 68.1% seropositive) and 2433 with early UA (67.8% female) were identified. DMARDs were initiated during the first month after the diagnosis to 92.0% of the patients with seropositive RA, 90.3% with seronegative RA and to 87.7% with UA (*p* < 0.001). Respectively, 72.1, 63.4, and 42.9% of the patients (*p* < 0.001) purchased methotrexate; 49.8, 35.9, and 16.0% (*p* < 0.001) as part of a DMARD combination during the first month. By the end of the first year after the diagnosis, self-injected biologics were purchased by 2.6, 5.3 and 3.1% (*p* < 0.001) of them. Only 1.4, 2.6 and 3.0% (*p* < 0.001) of the patients were not receiving any DMARDs. During the first year, 83.4% of the seropositive RA patients had purchased methotrexate, 50.4% sulfasalazine, 72.1% hydroxychloroquine, and 72.6% prednisolone.

**Conclusions:**

Currently, combination therapy including methotrexate is a common treatment strategy for early seropositive RA in Finland. Despite an easy access to biologics, these drugs are seldom needed during the first year after diagnosis.

## Background

All modern recommendations of drug therapy for rheumatoid arthritis (RA) underline the importance of early treatment aiming at remission and the key role of methotrexate (MTX). However, the role of the initial use of combinations of conventional synthetic disease modifying antirheumatic drugs (csDMARDs) causes dissension [1–3]. In Finland, the findings of the FIN-RACo and the NEO-RACo trials have influenced the clinical practice [4–7]. The national Current Care Guideline from the year 2015 advocates the initiation of three csDMARDs, the so-called FIN-RACo combination: MTX, sulfasalazine (SSZ), hydroxychloroquine (HCQ), and low-dose prednisolone (PRD) in early, active RA [8]. In the preceding versions in 2003 and 2009, however, the use of combination therapy was less rigorously recommended than in the current version.

Undifferentiated arthritis (UA) is an inflammatory arthritis where no specific diagnostic criteria are fulfilled. In Finland there are no specific early arthritis clinics, nor distinct treatment recommendations for UA, but the active treat-to-target (T2T) principle has been followed in clinical practice [9].

Implementing recommendations in real life may sometimes be suboptimal, but eventually they do bring about improvement to the use of DMARDs [10]. The data on the modern DMARD prescription patterns in early RA is still scarce, and seldom detailed. In a review article of studies done in 2002–2013, the penetration of antirheumatic therapy was found to be better in cohorts treated by rheumatologists (77–98%) than in cohorts allegedly treated by a mix of physicians (39–63%) [11]. In addition, a patient may not always use the prescribed medication. In the present study we describe how Finnish patients with early RA or UA purchased DMARDs between 2011 and 2015.

## Methods

Finland’s National Health Insurance covers both Finnish and foreign citizens residing permanently in Finland. The costs of most medicines prescribed by a doctor for the treatment of a disease are partially reimbursed by the Social Insurance Institution (SII) either at basic, lower special, or higher special rate, depending on the duration and severity of the disease. Patients with chronic inflammatory rheumatic disorders can be granted a special reimbursement (SR) (reimbursement of 65 to 72% of the drug price) for antirheumatic drugs after filling a medical certificate to SII. This certificate must describe the diagnostic procedures, an ICD10 diagnosis, and prescribed medication and be written in a rheumatology clinic. The certificates are reviewed by an insurance physician of the SII before the special reimbursement is granted after approximately 2–4 weeks. At one transaction, up to 3 months’ supply of medicines can be reimbursed. Since it is economically very much in the patients’ interest, practically all Finnish patients with anti-rheumatic medication for chronic inflammatory rheumatic disorders are entitled to reimbursement, and the pharmacists encourage their customers to request it if the reimbursement has not been applied for. There is also an annual maximum limit of out-of-pocket costs, which in 2020 is set at EUR 578. If the patients exceed the annual maximum, they can get an additional reimbursement, which means that for the rest of the year, they only pay a EUR 2.50 co-payment for each reimbursable medicine. This is of the greatest importance when the patient is prescribed some of the expensive medications such as self-injected biologics.

### Patient cohort

On grounds of the information in these medical certificates, SII maintains a nationwide register of the reimbursement decisions and the 3-digit ICD10 diagnoses behind them. From this register we assessed data collected between 1 January 2011 and 31 December 2014, and collected information on adult (> 16 years old) patients who, for the first time, had been granted a special reimbursement of medications for rheumatoid factor (RF) and/or anti-citrullinated peptide antibody (ACPA) positive (ICD-10 diagnosis M05) RA, RF and ACPA negative RA (M06), or UA (M13). The information included sex, date of birth, and the date of the reimbursement decision which is the index day in our study.

The SII maintains also a register on the drugs purchased from pharmacies and reimbursed according to the basic or the special rate. In this register, drugs are classified according to the Anatomical Therapeutic Chemical (ATC) code. The register also includes the amount of the drug and the date of purchase. From this register, we collected data on the drugs purchased by the study cohort for 31 days before the index day (to include medications possibly purchased before the reimbursement decision) and for up to 1 year after the index day. All csDMARDs, glucocorticoids and self-injected biologics were included in the analysis. The number of DMARD purchases by the end of the first year after the index date was evaluated in each diagnosis group, as well as among the patients who initiated the FIN-RACo combination (with or without PRD) or not. Our study does not include the small proportion of patients who have received intravenous biologicals in public hospitals at their cost, because these medications are not reimbursed and registered by the SII.

### Statistical methods

Statistical comparisons between diagnoses were made by using the χ2 test. Multivariate analyses were performed to identify whether age and gender were associated with the initiation of DMARD therapy (versus no DMARDs) or with the initiation of combination therapy (versus monotherapy) within the first month after the index date in each diagnosis group separately.

## Results

Between 2011 and 2014, altogether 9771 adult (> 16 years old) patients with a new inflammatory arthritis diagnosis were identified, 4998 patients with seropositive RA [67.0% female, mean (SD) age 58 (15) years]; 2340 with seronegative RA [67.7% female, 56 (17) years]; and 2433 with UA [67.8% female, age 49 (17) years].

The drugs purchased by the patients during the first month after the index day are presented in Table 1. The seropositive RA patients used more all, as well as MTX-based csDMARD-combinations, more MTX and prednisolone than the seronegative RA or UA patients. The use of any other csDMARDs than MTX, SSZ, or HCQ was rare during the first months in all patient groups, and only a handful of patients started using self-injected biological DMARDs (bDMARDs) at this early phase.

**Table 1:** Numbers and proportions (%) of patients with seropositive rheumatoid arthritis (RA), seronegative RA and undifferentiated arthritis using various anti-rheumatic drugs and drug combination strategies by the end of the first month after arthritis diagnosis

	Seropositive rheumatoid arthritis *N* = 4998	Seronegative rheumatoid arthritis *N* = 2340	Undifferentiated arthritis *N* = 2433	*p*-value
**Females N (%)**	3349 (67.0)	1584 (67.7)	1650 (67.8)	
**Mean age at diagnosis, years (SD)**	58 (15)	56 (17)	49 (17)	
**Combination strategies of DMARDs, N(%)**				
Two DMARDs	1684 (33.7)	727 (31.1)	395 (16.2)	< 0.001
Three DMARDs	1118 (22.4)	255 (10.9)	77 (3.1)	< 0.001
MTX-based combination	2489 (49.8)	841 (35.9)	390 (16.0)	< 0.001
FIN-RACo combination*	1114 (22.3)	253 (10.8)	75 (3.1)	< 0.001
MTX + SSZ + HCQ + PRD	922 (18.4)	205 (8.8)	52 (2.1)	
MTX + SSZ + HCQ	192 (3.8)	48 (2.1)	23 (0.9)	
**Medications**				
Methotrexate (MTX)	3602 (72.1)	1483 (63.4)	1043 (42.9)	< 0.001
MTX per os	3428 (68.6)	1399 (59.8)	949 (39.0)	
MTX s.c.	174 (3.5)	84 (3.6)	94 (3.9)	
Sulfasalazine (SSZ)	2059 (41.2)	880 (37.6)	1121 (46.1)	< 0.001
Hydroxychloroquine (HCQ)	2272 (55.5)	940 (40.2)	472 (19.4)	< 0.001
Leflunomide	51 (1.0)	30 (1.3)	23 (1)	0.48
Azathioprine	29 (0.6)	11 (0.5)	14 (0.6)	0.83
Aurathiomalate	9 (0.2)	2 (0.1)	2 (0.1)	0.44
Auranofin	3 (0.1)	1 (0.0)	1 (0.0)	0.95
Cyclosporine	1 (0.0)	3 (0.1)	6 (0.3)	0.015
Prednisolone (PRD)	3025 (60.5)	1352 (57.8)	890 (36.6)	< 0.001
Self-injected biologics (all)	41 (0.8)	28 (1.2)	17 (0.7)	0.15
Etanercept	3 (0.06)	11 (0.47)	2 (0.08)	
Adalimumab	4 (0.08)	6 (0.27)	1 (0.04)	
Certolizumab	1 (0.02)	0 (0)	0 (0)	
Golimumab	3 (0.03)	1 (0.04)	0 (0)	
Abatacept	1 (0.02)	0 (0)	0 (0)	
No antirheumatic medication	401 (8.0)	227 (9.7)	300 (13.3)	< 0.001
Only prednisolone	85 (1.7)	63 (2.7)	46 (1.9)	0.61

*FIN-RACo combination: Methotrexate (MTX), sulfasalazine (SSZ), and hydroxychloroquine (HCQ) often combined with low-dose prednisolone (PRD)

As expected, during the first year the proportions of patients purchasing any csDMARDs increased, especially in the seropositive RA group (Table 2). MTX, SSZ, and HCQ remained the absolute most used drugs, followed by leflunomide (LEF) with approximately 5% of patients in each group having purchased it during the first year; all other csDMARDs having a much smaller share. Self-injected biologics were initiated by 5.3% of the seronegative RA patients, and less often in the two other patient groups (*p* < 0.001). Only 3% of the patients in the UA group had not purchased any DMARDs during the first year, in the other groups the proportion of patients with no medications was even smaller (*p* < 0.001).

**Table 2:** Numbers and proportions (%) of patients with seropositive rheumatoid arthritis (RA), seronegative RA and undifferentiated arthritis having used various anti-rheumatic drugs by the end of the first year after arthritis diagnosis. Also, the number of DMARD purchases by the patients during the first year after diagnosis is shown

	Seropositive rheumatoid arthritis *N* = 4998	Seronegative rheumatoid arthritis *N* = 2340	Undifferentiated arthritis *N* = 2433	*p*-value
**Females N (%)**	3349 (67.0)	1584 (67.7)	1650 (67.8)	
**Mean age at diagnosis, years (SD)**	58 (15)	56 (17)	49 (17)	
**Number of DMARD purchases, median (IQR)**	11 (7, 16)	10 (6, 14)	7 (4, 11)	< 0.001
**Medications**				
Methotrexate (MTX)	4167 (83.4)	1789 (76.4)	1512 (62.1)	< 0.001
MTX per os	3998 (80.0)	1706 (72.9)	1406 (57.8)	
MTX s.c.	625 (12.5)	308 (13.2)	310 (12.7)	
Sulfasalazine (SSZ)	2520 (50.4)	1090 (46.6)	1362 (56.0)	< 0.001
Hydroxychloroquine (HCQ)	3603 (72.1)	1357 (58.0)	866 (35.6)	< 0.001
Leflunomide	256 (5.1)	121 (5.2)	119 (4.9)	0.89
Azathioprine	65 (1.3)	31 (1.3)	23 (0.9)	0.37
Aurathiomalate	42 (0.8)	12 (0.5)	8 (0.3)	0.023
Auranofin	4 (0.1)	2 (0.1)	2 (0.1)	0.99
Cyclosporine	13 (0.3)	10 (0.4)	23 (0.9)	< 0.001
Prednisolone (PRD)	3626 (72.6)	1706 (72.9)	1283 (52.7)	< 0.001
Self-injected biologics (all)	131 (2.6)	125 (5.3)	76 (3.1)	< 0.001
Etanercept	53 (1.1)	55 (2.4)	31 (1.3)	
Adalimumab	40 (0.8)	48 (2.1)	29 (1.2)	
Certolizumab	23 0.5)	19 (0.8)	12 (0.5)	
Golimumab	19 (0.4)	19 (0.8)	9 (0.4)	
Abatacept	8 (0.2)	5 (0.2)	1 (0.04)	
Tocilizumab	4 (0.1)	1 (0.04)	1 (0.04)	
Ustekinumab	0 (0)	0 (0)	1 (0.04)	
No antirheumatic medication	71 (1.4)	60 (2.6)	73 (3.0)	< 0.001
Only prednisolone	25 (0.5)	30 (1.3)	24 (1.0)	< 0.001

The median numbers (IQR) of patients’ DMARD purchases by the end of the first year after diagnosis are presented in Table 2. We further divided patients into two groups depending on whether they were initially treated with the FIN-RACo combination (MTX + SSZ + HCQ) or not. In the FIN-RACo group, the median (IQR) number of DMARD purchases was 18 (15 to 22) for seropositive RA, 19 (15 to 22) for seronegative RA, and 18 (15 o 24) for UA during the first year, whereas the respective numbers in the non-FIN-RACo group were 10 (6 to 13) for seropositive RA, 9 (6 to 13) for seronegative RA, and 7 (4 to 11) for UA. Thus, patients in the FIN-RACo group had almost twice as many DMARD purchases as the rest of the patients.

In multivariate analyses, using age and gender as covariates, we found that gender did not predict whether DMARDs were initiated or not, or whether the patient was treated with combination therapy or monotherapy during the first month after the index date in any of the three diagnosis groups. Higher age was negatively associated with DMARD initiation within a month from diagnosis among UA patients [OR 0.99 (CI 0.98 to 0.99)] but not among RA patients. The initiation of combination therapy decreased with a rising age among seropositive RA patients [OR 0.99 (CI 0.98 to 0.99)] but not among other diagnosis groups.

## Discussion

In Finland, early arthritis patients are mainly treated by rheumatologists. The Current Care Guideline advises general practitioners to refer all patients with suspected RA to specialist clinics. In addition, a rheumatologist’s certificate is needed to apply special reimbursement for antirheumatic medication, by which we identified our cases. Consequently, our cohort includes those arthritis patients, who had been examined by rheumatologists and prescribed DMARDs and glucocorticoids. Obviously, all patients with UA are not included. These facts explain, why within one month from the index date more than 90% of the RA patients purchased DMARDs and nearly 70% MTX. In seropositive patients, the percentages were higher, and within one year, only 1.4% of the seropositive and 2.6% of the seronegative RA patients had not purchased any DMARDs. For the UA patients the DMARD coverage was slightly less. These numbers also suggest a good drug adherence among Finnish arthritis patients.

Our results may not be comparable with studies in other settings. In a Canadian cohort studying the DMARD treatment of 24,942 early RA patients during the year following the diagnosis in 1997–2006, only 21% of patients treated by a general practitioner received any DMARDs, but 67% of those treated by a rheumatologist did so [12]. In a Danish cohort of 1516 early RA patients studying the initiation of MTX between 1996 and 2006, only 21% of the patients received MTX within 90 days; though, in 13% of the patients another DMARD had been initiated [13]. In studies based on large RA cohorts from US commercial and Medicare claims databases, the initiation of DMARD treatment within one year after diagnosis decreased from 63 to 56% between the cohorts 2004–08 and 2009–12 [14, 15]. Another US study based on claims databases found that over half of the 63,101 RA patients identified did not receive DMARD treatment within 90 days after diagnosis [16]. In a smaller Canadian cohort of 204 early RA patients in 2003–06, only 23% were prescribed a DMARD within 3 months and 47% within 6 months [17]. Also, in a recent Italian cohort of 1336 RA patients, less than 40% of the patients had started treatment with MTX within 3–6 months from the diagnosis [18].

Better coverages are found in contemporary materials treated by rheumatologists. In the French ESPOIR cohort of 775 early inflammatory arthritis patients, 77% received at least one DMARD after a median of four months [19]. A Canadian study of 339 RA patients found that 92% of the patients began DMARD therapy within three months [20]. In a multicenter ERAN cohort in UK and Eire, DMARDs were prescribed to 97% of the 808 early RA patients; however, the median time of DMARD initiation was 8 months after the symptom onset [21]. An Italian study reported that 83% of 10,401 patients were prescribed a csDMARD at RA diagnosis but only 6% of them received combination therapy [22].

Our previous analysis of Finnish early RA patients between 2000 and 2007 showed that although at the beginning of the study period SSZ was the most commonly prescribed DMARD during the first 3 months after the diagnosis, at the end of the observation period (2006–07) it had given way to MTX (69%) and combination DMARDs (53%) as the initial treatment [23]. Our earlier results also demonstrated that the proportion of patients starting triple combination within the first month increased from 6 to 16% between 2000 and 07. Our current results confirm that there has been a further increase. However, only 22% of early seropositive RA patients commenced the triple therapy recommended in the latest 2015 Current Care Guideline [8]. The Finnish recommendations from 2009 favored the start of the triple therapy and low dose prednisolone only for patients with very active RA, but not automatically as the first choice in all patients. Also, real life patients diagnosed with RA have seldom as active disease as patients in clinical trials [24], and further, they may have comorbidities and polypharmacy contraindicating certain medications, thus it is possible that rheumatologists base their decisions more on T2T principle than on slavishly following certain recommendations [25]. Either way, since we are lacking data of the levels of activity of the patients’ disease, further conclusions on whether only one-fifth of the patients had active disease requiring triple therapy cannot be drawn.

There is a distinction between guidelines encouraging a T2T strategy on the one hand and what actually happens in practice. In an ESPOIR cohort, where adherence to three of the EULAR recommendations concerning the start and early adjustment of DMARDs was studied, the adherence rate for all three recommendations was only 23% among early arthritis patients [26]. Still, among those patients whose treatment adhered to given recommendations, the risk of clinical and radiographic progression was lower. Knowing that the fulfilment of treatment guidelines in real life is always suboptimal, the strict national treatment recommendations, such as the recommendation of triple therapy initiation in early RA in Finland, will probably lead to optimal outcomes.

The number of DMARD purchases could reflect drug adherence although some patients may not always buy medication for the next three months as usually happens. In our analysis, the patients initiating the FIN-RACo combination had twice the number of DMARD purchases during the first year compared to those patients who did not start with the FIN-RACo combination. Frequent purchases suggest a regular drug usage and good survival rate of the FIN-RACo combination.

Two Finnish studies based on real life early arthritis patient cohorts have been published. The first one included 406 early RA or UA patients between 2008 and 11 [27]. Of the RA patients, at three months 20% were using triple therapy, 33% other MTX based combination, 36% MTX monotherapy, and 8% other DMARD monotherapy; for the UA patients the respective percentages were 6, 28, 43, and 17%, respectively. At one year, the proportions of RA patients using various medications had not changed markedly. In a more recent (2011–14) FIN-ERA cohort of 611 DMARD naïve early arthritis patients (506 RA and 105 UA patients) recruited in five Finnish outpatient rheumatology clinics, MTX-based combination therapy was initiated to 68% of the patients and the proportion of triple combination (MTX, SSZ and HCQ) was 31% [28]. These results, in line with our results, show that DMARD initiation for early arthritis patients is generally comprehensive in Finland.

In Finland there are no separate treatment recommendations for UA, but the active T2T principle is widely used in clinical practice regardless of the diagnosis. The European League of EULAR recommendations for early arthritis in 2007 recommended for patients at risk of developing persistent and/or erosive arthritis a DMARD as early as possible, preferably MTX, even if no classification criteria for a specific disease are fulfilled [29]. The latest update of the recommendation in 2016 presented no major changes to these principles [9]. The fulfillment of the EULAR recommendations for the treatment of early arthritis was studied in 813 patients from the ESPOIR cohort between 2002 and 05; 78% of patients started a DMARD, 67% MTX and 52% reached remission [30]. In our material the DMARD initiation was more comprehensive, but the proportion on MTX lower; this might be explained by the fact that traditionally SSZ has been prescribed in seronegative oligoarthritis in Finland.

In our study, the use of self-injected biological DMARDs was more common among seronegative RA patients than seropositive RA or UA patients. Seronegative RA may be a heterogenous group of diseases, as shown in a recent 10-year observational study [31], thus explaining poorer treatment outcomes with csDMARDs and a greater need for switching to bDMARDs.

A shortcoming of this study is the lack of clinical data; we do not have information of the disease’s activity at diagnosis, nor at follow-up. Further, we may miss patients that the physician intends not to treat. Nevertheless, the incidence of seropositive RA has remained stable throughout this millennium, that of seronegative RA has decreased slightly supposedly due to changed diagnostics, and for the same reason, the incidence of UA has increased [32]. Thus, it seems unlikely that we are missing many patients. However, since this is a register-based study and we lack the data on the duration of symptoms before the diagnosis, it is possible that the current study includes some patients that are diagnosed with a time lag; in these cases, DMARDs used represent patients’ initial treatment rather than the treatment of early rheumatic disease. Also, we do not know how high a proportion of UA patients received a more specific diagnosis later; this could offer an interesting area for further research. Further, in the lack of clinical data it is possible that a certain proportion of the UA patients in our study may not be comparable to patients in so called early arthritis clinics, but have a chronic inflammatory arthritis requiring specific anti-rheumatic drug therapy.

Even though we do not have any clinical outcome measures, the initiation of self-injected biologics served as a surrogate marker of treatment failure. We were expecting to see that patients having received combination DMARDs as their first treatment, and thus judged by their treating rheumatologist to have an active disease to be the ones to end up starting a biologic earlier and more often than other patients, but at least during the first year that was not the case. Thus, at least in the early phase, combination DMARD treatment appears to be effective.

Although we are lacking the data on infusion based biological drugs, only a small proportion of patients are initiating them during the first year after the diagnosis, the time period of interest in our study. According to the Finnish ROB-FIN register study, in 2004–14 the first TNF-inhibitors were initiated to RA patients after a median (IQR) of 8.2 (2.4–17) years of disease duration, most often with adalimumab (39%) or etanercept (39%), while infliximab was a rarer choice (12%) [33]. According to the most recent, yet unpublished, ROB-FIN register data from 2010 to 15, the use of infliximab as the first biologic had decreased from 7.5 to 4.2% for RA patients (Kalle Aaltonen, personal communication). Rituximab was the first choice for as many as 20–23% of the RA patients. Still, the majority started self-injected drugs, and the median (IQR) point of starting the first biologic was after 10 (4.4–18) years of disease duration. Consequently, our results of the first year treatment would hardly have changed markedly, were the infusion-based drugs included.

## Conclusions

In this study we wanted to describe the drug therapies used for early rheumatoid (RA) and undifferentiated (UA) arthritis patients between 2011 and 2015. The rheumatologist-based treatment received by the Finnish new-onset arthritis patients is early initiation of cDMARDs, mainly MTX, and often in combinations.

## Data Availability

The data that support the findings of this study are available from the Social Insurance Institution (SII) of Finland but restrictions apply to the availability of these data, which were used under license for the current study, and so are not publicly available. Data are however available from the authors upon reasonable request and with permission of the SII.

## Abbreviations

ACPA: Anti-citrullinated peptide antibody
ATC: Anatomical Therapeutic Chemical
bDMARDs: Biological DMARDs
csDMARD: Conventional synthetic disease modifying antirheumatic drug
EULAR: European League Against Rheumatism
FIN-RACo: The Finnish Rheumatoid Arthritis Combination Therapy Trial
HCQ: Hydroxychloroquine
LEF: Leflunomide
MTX: Methotrexate
PRD: Prednisolone
RA: Rheumatoid arthritis
RF: Rheumatoid factor
ROB-FIN: The National Register for Biologic Treatment in Finland
SII: Social Insurance Institution
SR: Special reimbursement
SSZ: Sulfasalazine
UA: Undifferentiated arthritis
T2T: Treat-to-target
